# Transcriptional profiling of mammary gland in Holstein cows with extremely different milk protein and fat percentage using RNA sequencing

**DOI:** 10.1186/1471-2164-15-226

**Published:** 2014-03-24

**Authors:** Xiaogang Cui, Yali Hou, Shaohua Yang, Yan Xie, Shengli Zhang, Yuan Zhang, Qin Zhang, Xuemei Lu, George E Liu, Dongxiao Sun

**Affiliations:** 1 Key Laboratory of Animal Genetics and Breeding of Ministry of Agriculture, National Engineering Laboratory of Animal Breeding, College of Animal Science and Technology, China Agricultural University, Beijing 100193, China; 2 Laboratory of Disease Genomics and Individualized Medicine, Beijing Institute of Genomics, Chinese Academy of Sciences, Beijing 100029, China; 3 Bovine Functional Genomics Laboratory, ANRI, USDA-ARS, Beltsville, MD 20705, USA

**Keywords:** Transcriptome, Differentially expressed gene, Mammary gland, Protein percentage, Fat percentage, RNA-Seq, Dairy cattle

## Abstract

**Background:**

Recently, RNA sequencing (RNA-seq) has rapidly emerged as a major transcriptome profiling system. Elucidation of the bovine mammary gland transcriptome by RNA-seq is essential for identifying candidate genes that contribute to milk composition traits in dairy cattle.

**Results:**

We used massive, parallel, high-throughput, RNA-seq to generate the bovine transcriptome from the mammary glands of four lactating Holstein cows with extremely high and low phenotypic values of milk protein and fat percentage. In total, we obtained 48,967,376–75,572,578 uniquely mapped reads that covered 82.25% of the current annotated transcripts, which represented 15549 mRNA transcripts, across all the four mammary gland samples. Among them, 31 differentially expressed genes (*p < 0.05*, false discovery rate *q* < 0.05) between the high and low groups of cows were revealed. Gene ontology and pathway analysis demonstrated that the 31 differently expressed genes were enriched in specific biological processes with regard to protein metabolism, fat metabolism, and mammary gland development (*p < 0.05*). Integrated analysis of differential gene expression, previously reported quantitative trait loci, and genome-wide association studies indicated that *TRIB3*, *SAA* (*SAA1*, *SAA3*, and *M-SAA3.2*), *VEGFA, PTHLH*, and *RPL23A* were the most promising candidate genes affecting milk protein and fat percentage.

**Conclusions:**

This study investigated the complexity of the mammary gland transcriptome in dairy cattle using RNA-seq. Integrated analysis of differential gene expression and the reported quantitative trait loci and genome-wide association study data permitted the identification of candidate key genes for milk composition traits.

## Background

In dairy cattle, milk yield and milk components are two of the most important economic traits. DNA-based marker-assisted selection has certain advantages over the traditional dairy cattle breeding programs, such as shortening of the generation interval and enhancing the selection accuracy of young bulls, by incorporating detected quantitative trait loci (QTLs) into the genetic evaluation. Therefore, since the first report by Georges et al. [1], extensive QTL mapping has been implemented to detect QTLs with large effects on milk production traits. As of August 23, 2013, 422, 823, and 648 QTLs for milk yield, milk protein, and milk fat have been reported via marker-QTL linkage analysis (LA) and/or linkage disequilibrium (LD) ( http://www.animalgenome.org/cattle/). Most recently, as a powerful tool for revealing the genetic basis of complex traits, several genome-wide association studies (GWASs) were conducted to map QTLs for milk yield and composition traits in dairy cattle [2-11]. To date, these two approaches have detected a large number of significant chromosome segments and genetic associations with milk yield and composition, which include two confirmed causal mutations: *DGAT1*p.Lys232Ala and *GHR* p.Phe279Tyr [2-13].

Besides genetic data, gene expression profiles and metabolic pathway analysis offer new opportunities to elucidate the underlying mechanisms of complex traits in humans and farm animals. Recently, along with the rapid development and cost reduction of next generation sequencing (NGS), sequence-based assays of transcriptomes, namely RNA sequencing (RNA-seq), have become a comprehensive and accurate tool for gene expression pattern analyses. As compared with microarray technology, RNA-seq enables analysis of the complexity of whole eukaryotic transcriptomes with less bias, greater dynamic range, lower frequency of false-positive signals, and higher reproducibility [14,15]. There have been several studies on the bovine transcriptome using RNA-seq techniques, such as the bovine embryo and milk transcriptome [16-21]; however, no studies on the bovine mammary gland transcriptome by RNA-seq have been published. Two studies on the transcriptome of the mammary gland of Holstein cows using an oligonucleotide microarray have been presented, one of which compared the gene expression profile before (dry) and after (milk) parturition, using an Affymetrix cDNA array [22]. The other performed functional analyses of differentially expressed gene patterns across -30, -15, 1, 15, 30, 60, 120, 240, and 300 days relative to parturition with microarray [23]. In the present study, we used RNA-seq technology to examine the genome-wide gene expression profile in mammary glands between two groups of Holstein cows with extremely high and low milk protein percentage (PP) and fat percentage (FP). We then integrated the analysis of the differentially expressed genes detected in this work and the previously reported QTLs and GWAS data to identify key genes affecting milk protein and FP.

## Methods

### Animals and mammary gland tissue sample collection

Four lactating Chinese Holstein cows in their 2nd/3rd lactation, from different families, were selected from among 30,000 Holstein cows fed in the Beijing Sanyuanlvhe Dairy Farming Center. The selection was based on their monthly test-day milk PP and FP records for the current lactation and previous lactation(s), which were provided by the Dairy Data Center of China. The average PP and FP was 3.1% (2.7–3.8%) and 3.6% (3.1–4.5%) in this population. To keep the environment factors identical, four cows that were in almost the same period of lactation (353, 341, 377, and 325 days in milk of the 2nd, 3rd, 2nd, and 3rd lactations, respectively) and collected from the same farm, which possesses 800 Holstein cows in total. According to the last PP and FP record of the current lactation, four cows were divided into two groups with extremes of the phenotypic values for PP and FP: two cows (**high group**) had high PP (3.6% and 3.8%) and FP (3.9% and 4.5%); the other two cows (**low group**) showed low PP (3.0%, 2.9%) and FP (3.2%, 3.1%).

The cows were killed by electroshock, bled, skinned, and dismembered in the same slaughterhouse. The rear mammary gland from each individual was removed within 30 min after slaughter. The right rear quarter of the mammary gland was cut in half lengthways from the teat such that white mammary ducts and pink epithelium tissue were clearly observed and some milk flowed out. Five pieces of epithelium tissue samples per cow were carefully collected for RNA isolation, placed into a clean RNAse-free Eppendorf tube, and stored in liquid nitrogen. All sample collection procedures were carried out in strict accordance with the protocol approved by the Animal Welfare Committee of China Agricultural University (Permit Number: DK996).

Considering the large genetic effect of the *DGAT1* gene on milk composition traits, these four cows were genotyped on the *DGAT1*p.Lys232Ala mutation. A pair of primers [24] was used to amplify a 201-bp fragment comprising *DGAT1* p.Lys232Ala. Forty microliters of polymerase chain reaction products of each individual were directly sequenced using an ABI3730XL sequencer (Applied Biosystems, CA, USA). As a result, the genotypes of the cows with high PP and FP and cows with low PP and FP on *DGAT1*p.Lys232Ala were identified to be KA, KA, KA, and AA, respectively (Additional file 1: Figure S1). The allele K has been reported to be associated with increased FP and PP [25,26].

### RNA isolation and quality assessment

The TRIzol reagent (Invitrogen, Carlsbad, CA) was used to extract total RNA from mammary epithelium samples. RNase-free DNase I (New England Biolabs) was used to remove DNA contamination from the RNA by incubating for 30 min at 37°C. One piece of the five samples from each cow was randomly chosen for RNA isolation. The average RNA yield from each sample was around 73 μg from 100 mg of tissue. RNA degradation and contamination was monitored on 1% agarose gels. A NanoPhotometer® spectrophotometer (Implen, CA, USA) was used to check RNA purity. A Qubit® RNA Assay Kit in Qubit® 2.0 Fluorometer (Life Technologies, CA, USA) measured the RNA concentration and the RNA Nano 6000 Assay Kit of the Bioanalyzer 2100 system (Agilent Technologies, CA, USA) assessed the RNA integrity.

### Transcriptome sample preparation, clustering, and re-sequencing

A total of 3 μg of RNA per sample per cow was used as the input material for the RNA sample preparations. The RNA integrity number (RIN) values of the two samples from cows with high milk PP and FP and two samples from the cows with low PP and FP were 7.0, 7.5, 7.2, and 7.5, respectively. Sequencing libraries were generated using IlluminaTruSeq™ RNA Sample Preparation Kit (Illumina, San Diego, CA, USA), following manufacturer’s recommendations. Four index codes were added to attribute sequences to each sample. Briefly, poly-T oligo-attached magnetic beads purified the mRNA from total RNA. Divalent cations under elevated temperature in an Illumina proprietary fragmentation buffer then fragmented the mRNAs. Random oligonucleotides and SuperScript II were used to synthesize first-strand cDNA. DNA polymerase I and RNase H subsequently generated second-strand cDNA. Exonuclease/polymerase activities were used to convert remaining overhangs to blunt ends and to remove enzymes. After adenylation of 3′ ends of DNA fragments, Illumina PE adapter oligonucleotides were ligated to prepare for hybridization. To select cDNA fragments approximately 200 bp in length, the library fragments were purified with AMPure XP system (Beckman Coulter, Beverly, USA). An Illumina polymerase chain reaction (PCR) Primer Cocktail in a 10-cycle PCR reaction selectively enriched DNA fragments with ligated adaptor molecules on both ends. The AMPure XP system purified the products, which were quantified using the Agilent high-sensitivity DNA assay on the Agilent Bioanalyzer 2100 system.

A cBot Cluster Generation System, using TruSeq PE Cluster Kit v3-cBot-HS (Illumina) according to the manufacturer’s instructions, clustered the index-coded samples. After cluster generation, an Illumina Hiseq 2000 platform sequenced the libraries, generating around 268 million 100-bp paired-end reads in total.

### Quality control for paired-end reads

Raw data (raw reads) formatted as fastq were processed through our self-written perl scripts. In this step, clean data (clean reads) were obtained by removing reads containing adapters, reads containing at least 10 Ns, and low-quality reads (more than half of the reads having a phred base quality score of less than 5) from the raw data. After that, the description statistics for the clean data, such as Q20 (the proportion of bases with a phred base quality score greater than 20; i.e., the proportion of read bases whose error rate is less than 1%), Q30 (the proportion of bases with a phred base quality score greater than 30; i.e., the proportion of read bases whose error rate is less than 0.1%), GC content, and sequence duplication level of the clean data were calculated (Additional file 2: Table S1). All the downstream analyses were based on the clean data.

### Reads alignment to the bovine reference genome and annotated transcripts

The Cattle reference genome (UMD3.1.66) and corresponding gene model annotation files were downloaded directly from the cow genome website ( ftp://ftp.ensembl.org/pub/release-69/fasta/bos_taurus/dna/). An index of the reference genome was built using Bowtie v0.12.8 and paired-end clean reads for each individual were aligned to the reference genome by TopHat v2.0.0 ( http://tophat.cbcb.umd.edu/). The detailed alignment information is presented in Additional file 2: Table S1, including total numbers of reads, mapped reads, and unique mapped reads.

### Identification of differentially expressed genes

The numbers of RNA-seq reads produced from a transcript is directly proportional to its abundance; therefore, the gene expression level could be quantified by the reads count. Commonly used Cuffdiff [27] and DESeq [28] methods identified the differentially expressed genes between the different groups.

For Cuffdiff, the commonly used fragments per kilobase of transcript per million mapped fragments (FPKM) value [29] in pair-end sequencing experiments incorporated two normalization steps; i.e., the number of fragments was normalized by the transcript’s length and the total yield of the fragments to ensure accurate quantification of the gene’s expression [27]. TopHat’s read alignments were assembled by Cufflinks [27], and then the differentially expressed genes and transcripts across Holstein cows with high and low PP and FP were detected and quantified by Cuffdiff, which is included in the Cufflinks package, using a rigorous sophisticated statistical analysis [27]. Negative binomial distribution was introduced to address the gene expression (counts) modeling for single-isoform genes and a mixed model of negative binomials using the beta distribution parameters as the mixture weights, followed by a t-test for defined statistics, to identify the significantly differentially expressed genes [30]. The differentially expressed genes, their corresponding attributes, fold changes (in log_2_ scale), *p*-values, and *q*-values (false discovery rate corrected *p* values) were reported in the output files from Cuffdiff. The significance of the gene expression difference was determined as yes if the *p*-value was less than the false discovery rate after Benjamini-Hochberg correction for multiple testing ( http://cufflinks.cbcb.umd.edu/manual.html). In addition, to filter out those alignments that lie within intronic intervals implied by the spliced alignments, we set the pre-mrna-fraction as 0.25.

For the DESeq method, differentially expressed genes were detected by using the Deseq R package (1.8.3). DESeq allows the accurate comparisons between extreme cow groups by normalizing the number of reads, which accommodates a scaling factor for a given cow by computing the median of the ratio, for each gene, of its read count over its geometric mean across all cows [30]. Negative binomial distribution was introduced to address the gene expression (counts) modeling, and the Fisher’s exact test was adopted to test the significantly differential expressed genes. The *p*-values were adjusted using the Benjamini and Hochberg method. A corrected *p*-value of 0.05 was set as the threshold for significant differential expression.

### GO and gene functional analysis of differentially expressed genes

The Goseq R package implemented gene ontology (GO) enrichment analysis of the differentially expressed genes, in which gene length bias was corrected. GO terms with corrected *p*-values less than 0.05 were considered significantly enriched among the differential expressed genes.

In addition, Ingenuity Pathways Analysis (IPA) software v9.0 ( http://www.ingenuity.com/, Ingenuity Systems, Redwood City, CA) was used to analyze the differentially expressed genes between cows with high and low PP and FP. The accessions of these genes were imported into IPA, then the "Core Analysis" function included in IPA was used to analyze the genes in the context of networks, biological functions, and canonical pathways. The detailed information concerning IPA analyses was published previously [31,32].

### Real-time quantitative reverse-transcription-PCR (qRT-PCR)

To validate the repeatability and reproducibility of gene expression data obtained by RNA sequencing in Holstein cows, qRT-PCR was carried out on 11 randomly selected differentially expressed genes using the total RNA used for RNA-seq. Superscript II reverse transcriptase (Invitrogen, Carlsbad, CA) synthesized first-strand cDNA. Primer3 ( http://fokker.wi.mit.edu/primer3/input.htm) designed gene-specific primers, which were validated using Oligo 6.0. Primer sequences are shown in Additional file 3: Table S2. The mRNA levels of the differentially expressed genes were normalized against two housekeeping genes, *GAPDH* and *ACTB*, in the corresponding samples. qRT-PCR was carried out in triplicate with the DyNAmo SYBR Green PCR kit (Applied Biosystems Inc.) on a LightCycler480 (Roche Applied Science, Penzberg, Germany), using the following program: 95°C for 10 min; 45 cycles of 95°C for 10 s, 60°C for 10 s, and 72°C for 10 s; 72°C for 6 min.

## Results

### Sequencing and mapping of the bovine mammary gland transcriptome

We sequenced cDNA libraries of four mammary epithelium samples from two Holstein cows with higher milk PP and FP and two with lower phenotypic values. In total, we acquired 53,294,906–83,019,642 paired-end reads of 100 bp in length per sample. As a result, the total read length was 26.6 gigabases (Gb) for the four samples. Alignment of the sequence reads against the bovine genome UMD3.1.66 yielded 90.6–91.9% of uniquely aligned reads across the four samples, of which 80–85% fell in annotated exons, 4–6% were located in introns, and the remaining 11–14% was assigned to intergenic regions. Unmapped or multi-position matched reads (8.1–9.4%) were excluded from further analyses (Additional file 2: Table S1). Consequently, 15549 mRNA transcripts were detected as expressed in the four mammary gland samples, among which there were certain well-known genes affecting milk traits; e.g., *K-casein* (*CSN3*), *β-casein* (*CSN2*), *a-lactalbumin* (*LALBA*), *β-lactoglobulin* (*BLG*), *DGAT1*, *GHR*, *STAT5A*, *STAT5B*, and *SCD*. These results further confirmed the reliability of RNA-seq and the sampling accuracy of the mammary gland tissue used in this study. Furthermore, using a pairwise approach, two cows in the same group were used to eliminate the background noise of individual-specific transcription, enabling acquisition of more relevant data from the two groups. The correlation coefficient (R^2^) between the two individuals within the high and low groups for milk PP and FP was calculated based on the fragments per kilobase of transcript per million mapped fragment (FRPM value) of each cow and was shown to be 0.986 and 0.983, respectively, indicating that the similarity of the two biological replicates within each group was sufficiently high (Additional file 4: Figure S2).

### Differential gene expression between high and low groups for milk PP and FP

**Figure 1:**
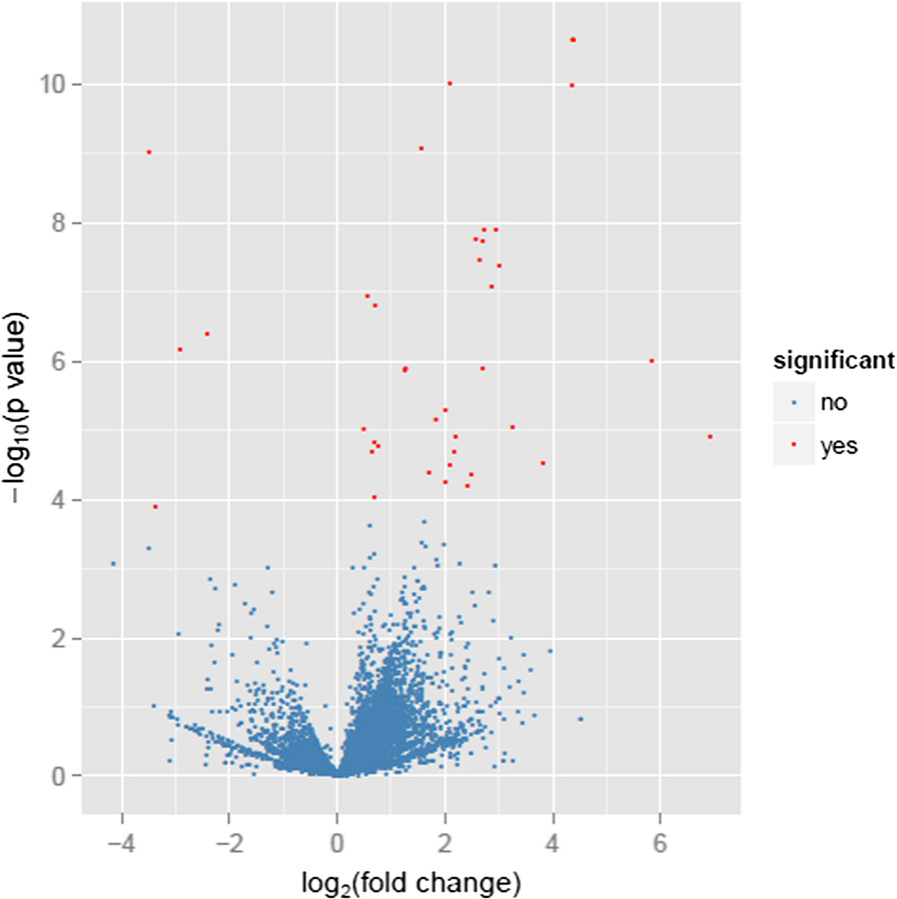
**Volcano plot displaying differential expressed genes between the mammary glands of two cows with high milk PP and FP and two cows with low PP and FP.** The *y*-axis corresponds to the mean expression value of log_10_ (*p*-value), and the *x*-axis displays the log2 fold change value. The red dots represent the significantly differentially expressed transcripts (*p* < 0.05, false discovery rate (FDR) *q* < 0.05) between two cows with high milk PP and FP and two cows with low PP and FP; the blue dots represent the transcripts whose expression levels did not reach statistical significance (*p* > 0.05, FDR *q* > 0.05) between the high and low groups.

Using DEseq and Cuffdiff methods, the differential gene expression profile between the Holstein cows with the higher and lower PP and FP was examined. In total, 31 top half expressed genes were detected as significantly different by DEseq, among which six genes were also identified by Cuffdiff (*p < 0.05*, FDR *q* < 0.05). The expression levels of 28 of the 31 genes were downregulated in the cows with higher PP and FP; the other three genes showed higher expression in the cows with lower PP and FP. Details of differentially expressed genes, their full names, as well as *p-* and *q*-values, are shown in Table 1 and Figure 1.

**Table 1:** Thirty-one differentially expressed transcripts between the mammary gland of two cows with high milk protein and fat percentage and two cows with low protein and fat percentage

**Transcript symbol**	**NCBI gene ID**	**Description**	**log**_**2 **_**fold change (high/low)**	**p value**	**FDR q value**	**Detected by**
*SAA3*	281474	Serum amyloid A3	-2.09	9.90E-11	1.47E-07	DEseq
*HEATR7B2*	518808	HEAT repeat family member 7B2	3.49	8.36E-10	1.04E-06	DEseq
*C4BPA*	281651	Complement component 4 binding protein, alpha	-1.57	9.20E-10	1.04E-06	DEseq
*TRIB3*	538465	Tribbles homolog 3	-2.64	3.38E-08	2.63E-05	DEseq, Cuffdiff
*SESN2*	509863	Sestrin 2	-3.01	4.08E-08	2.99E-05	DEseq, Cuffdiff
*CHAC1*	505991	Cation transport regulator homolog 1	-2.86	8.62E-08	5.97E-05	DEseq, Cuffdiff
*HSPD1*	511913	Heat shock 60 kD protein 1	-0.57	1.12E-07	7.35E-05	DEseq
*SLC25A38*	512325	Solute carrier family 25 member 38	-0.70	1.57E-07	9.80E-05	DEseq
*NR4A1*	528390	Nuclear receptor subfamily 4, group A, member 1	2.42	4.10E-07	0.000243	DEseq
*BMX*	531514	BMX non-receptor tyrosine kinase	2.92	6.94E-07	0.000393	DEseq
*SAA1*	616035	Serum amyloid A1	-5.84	9.99E-07	0.000541	DEseq, Cuffdiff
*ATF3*	515266	Activating transcription factor 3	-2.70	1.24E-06	0.000641	DEseq, Cuffdiff
*RPL23A*	507168	Ribosomal protein L23a	-5.31	1.88E-05	0.038988	DEseq, Cuffdiff
*CDH16*	508777	Cadherin 16, KSP-cadherin	-1.27	1.29E-06	0.000641	DEseq
*VEGFA*	281572	Vascular endothelial growth factor A	-1.25	1.35E-06	0.000647	DEseq
*KRT24*	788424	Keratin 24	-1.83	6.96E-06	0.003095	DEseq
*EIF4G3*	534618	Eukaryotic translation initiation factor 4 gamma, 3	-0.49	9.76E-06	0.004052	DEseq
*CDKN1A*	513497	Cyclin-dependent kinase inhibitor 1A	-2.20	1.22E-05	0.004742	DEseq
*BOLA-DQB*	282495	Major histocompatibility complex, class II, DQ beta	-6.92	1.21E-05	0.004742	DEseq
*ARID1B*	505229	AT rich interactive domain 1B (SWI1-like)	-0.70	1.46E-05	0.005502	DEseq
*PTHLH*	286767	Parathyroid hormone-like hormone	-0.76	1.69E-05	0.006194	DEseq
*ZC3H14*	511473	Zinc finger CCCH-type containing 14	-0.65	1.99E-05	0.007094	DEseq
*H4*	280691	Histone H4	-2.17	2.07E-05	0.007179	DEseq
*FAM71A*	616153	Family with sequence similarity 71, member A	-1.0	2.53E-05	0.008504	DEseq
*THBS4*	541281	Thrombospondin 4	-2.10	3.21E-05	0.010249	DEseq
*DDIT3*	777788	DNA-damage-inducible transcript 3	-1.70	4.01E-05	0.012494	DEseq
*M-SAA3.2*	618238	Mammary serum amyloid A3.2	-2.49	4.39E-05	0.013339	DEseq
*HIST1H2AC*	506900	Histone cluster 1, H2ac	-2.01	5.54E-05	0.016423	DEseq
*FCAMR*	100297408	Fc receptor, IgA, IgM, high affinity	-2.42	6.43E-05	0.018621	DEseq
*DNER*	516825	Delta/notch-like EGF repeat containing	-1.0	8.29E-05	0.023475	DEseq
*P4HA2*	507327	Prolyl 4-hydroxylase, alpha polypeptide II	-0.69	9.24E-05	0.025587	DEseq

Furthermore, we randomly selected 11 differentially expressed genes, including *SAA3, TRIB3*, *SESN2, CHAC1*, *SAA1, ATF3*, *ARID1B*, *PTHLH*, *ZC3H14*, *H4,* and *DDIT3*, to perform expression patterns validation using qRT-PCR. For these selected 11 genes, the correlations between the mRNA expression level from qRT-PCR and RNA-seq were relatively high, with correlation coefficients of 0.81 and 0.82, which were normalized by housekeeping genes *GAPDH* and *ACTB*, respectively, confirming the high reproducibility of RNA-seq data in this study (Additional file 5: Figure S3).

### Gene Ontology enrichment and pathway analysis

To further investigate the functional associations of the 31 genes differentially expressed genes, we performed the gene ontology (GO) analysis at the GO database ( http://www.geneontology.org/GO.database.shtml). Several significant GO categories were enriched (*p* < 0.01), including GO processes related to protein and lipid metabolism, mammary gland development, differentiation, and immune function. Such biological processes were involved in high-density lipoprotein particles, export of cholesterol, protein refolding, ubiquitin-protein ligase regulator activity, angiogenesis of vascular endothelial and epithelial cell, cell viability/death/differentiation, mammary gland bud elongation, lipopolysaccharide receptor complex, regulation of MAP kinase activity, and activation of immune-related cells (Table 2).

**Table 2:** Summary of the GO analysis of 31 differentially expressed genes between the mammary gland of two cows with high milk protein and fat percentage and two cows with low protein and fat percentage

**GO ID**	**GO term**	**No. of differentially expressed genes**	**P-value**
GO:0034364	High-density lipoprotein particle	4	3.26E-06
GO:0006953	Acute-phase response	4	3.26E-06
GO:0001938	Positive regulation of endothelial cell proliferation	3	3.50E-05
GO:0035767	Endothelial cell chemotaxis	2	5.11E-05
GO:0043129	Surfactant homeostasis	3	0.000120
GO:0002042	Cell migration involved in sprouting angiogenesis	2	0.000126
GO:0035924	Cellular response to vascular endothelial growth factor stimulus	2	0.000224
GO:0007050	Cell cycle arrest	4	0.000321
GO:0043498	Cell surface binding	3	0.000347
GO:0030855	Epithelial cell differentiation	3	0.001532
GO:2000016	Negative regulation of determination of dorsal identity	1	0.001848
GO:0019912	cyclin-dependent protein kinase activating kinase activity	1	0.001848
GO:0001791	IgM binding	1	0.001848
GO:0002575	Basophil chemotaxis	1	0.001848
GO:0043184	Vascular endothelial growth factor receptor 2 binding	1	0.001848
GO:0005576	Extracellular region	9	0.002867
GO:0043433	Negative regulation of sequence-specific DNA-binding transcription factor activity	3	0.003109
GO:2000278	Regulation of DNA biosynthetic process	1	0.003694
GO:0070557	PCNA-p21 complex	1	0.003694
GO:0043183	Vascular endothelial growth factor receptor 1 binding	1	0.003694
GO:0002368	B-cell cytokine production	2	0.003694
GO:0030135	Coated vesicle	2	0.003694
GO:0048291	Isotype switching to IgG isotypes	2	0.003694
GO:0019862	IgA binding	1	0.003694
GO:0060649	Mammary gland bud elongation	2	0.003694
GO:0060659	Nipple sheath formation	2	0.003694
GO:0060319	Primitive erythrocyte differentiation	1	0.003694
GO:0042789	mRNA transcription from RNA polymerase II promoter	1	0.003694
GO:0010827	Regulation of glucose transport	1	0.003694
GO:0005172	Vascular endothelial growth factor receptor binding	1	0.003704
GO:0051451	Myoblast migration	1	0.003712
GO:0050731	Positive regulation of peptidyl-tyrosine phosphorylation	2	0.004336
GO:0002842	Positive regulation of T-cell-mediated immune response to tumor cell	2	0.005536
GO:0042026	Protein refolding	2	0.005536
GO:0033158	Regulation of protein import into nucleus, translocation	1	0.005537
GO:0055106	Ubiquitin-protein ligase regulator activity	1	0.005537
GO:0071603	Endothelial cell-cell adhesion	1	0.005554
GO:0015204	Urea transmembrane transporter activity	2	0.005573
GO:0071918	Urea transmembrane transport	2	0.005573
GO:0018401	Peptidyl-proline hydroxylation to 4-hydroxy-L-proline	1	0.005604
GO:0030141	Stored secretory granule	4	0.006394
GO:0002755	MyD88-dependent toll-like receptor signaling pathway	2	0.007375
GO:0015840	Urea transport	2	0.007376
GO:0005372	Water transmembrane transporter activity	2	0.007376
GO:0090050	Positive regulation of cell migration involved in sprouting angiogenesis	1	0.007379
GO:0050927	Positive regulation of positive chemotaxis	1	0.007385
GO:0060754	Positive regulation of mast cell chemotaxis	1	0.007434
GO:0031077	Post-embryonic camera-type eye development	1	0.007435
GO:0043117	Positive regulation of vascular permeability	1	0.007439
GO:0010740	Positive regulation of intracellular protein kinase cascade	1	0.007509
GO:0008201	Heparin binding	2	0.007540
GO:0007220	Notch receptor processing	1	0.007634
GO:0005769	Early endosome	3	0.008615
GO:0046696	Lipopolysaccharide receptor complex	2	0.009213
GO:0043405	Regulation of MAP kinase activity	1	0.009217
GO:0051428	Peptide hormone receptor binding	2	0.009221
GO:0032793	Positive regulation of CREB transcription factor activity	1	0.009358

At the same time, we also performed metabolic pathway analysis on the 31 differentially expressed genes using Ingenuity Pathways Analysis (IPA) software v 9.0. A significant molecular function involving "lipid metabolism" was enriched. Three genes, *TRIB3*, *SAA1*, and *ATF3*, were found to be involved in the lipid metabolism pathway (*p* < 0.05), which is mostly related to the formation of milk fat traits. The detailed list of the genes and their functional annotations are shown in Table 3.

**Table 3:** Gene function and pathway analysis of 31 differentially expressed genes between the mammary gland of two cows with high milk protein and fat percentage and two cows with low protein and fat percentage with IPA

**Pathway**	**Functional annotation**	**p-value**	**Gene**
Lipid metabolism	Export of cholesterol	3.36e-04	*SAA1*
Lipid metabolism	Accumulation of beta-estradiol	6.73e-04	*SAA1*
Lipid metabolism	Concentration of bile acid	6.38e-03	*ATF3*
	Apoptosis of epithelial cell lines		
	Cell death of connective tissue cells		
	Cell death of epithelial cells		
	development of endothelial cell lines		
Lipid metabolism	Quantity of steroid	8.53e-03	*ATF3, SAA1*
Lipid metabolism	Fatty acid metabolism	1.25e-02	*TRIB3, SAA1*
Lipid metabolism	Efflux of cholesterol	2.27e-02	*SAA1*
Lipid metabolism	Homeostasis of cholesterol	2.37e-02	*SAA1*
Lipid metabolism	Metabolism of cholesterol	3.16e-02	*SAA1*

### Integrated analysis of RNA-seq, QTL, and GWAS data

To gain further insights into the association of the 31 differentially expressed genes with milk composition traits, we integrated the 31 genes and previously reported QTL mapping and GWAS data by comparing their chromosome positions with those of the QTLs ( http://www.animalgenome.org/cgi-bin/QTLdb) and the significant SNPs detected by GWAS for milk production traits. The positions of the 31 genes on the chromosomes and detailed information about the nearest QTLs and significant SNPs are shown in Tables 4 and 5.

**Table 4:** Detailed information on the reported QTLs containing the 31 differentially expressed genes between the mammary gland of two cows with high milk protein and fat percentage and two cows with low protein and fat percentage

**Gene name**	**Position (bp)**	**Position (cM)**^**1**^	**Previously reported QTL**			
			**Distance to QTL peak (cM)**	**CI and peak location (cM)**	**Trait**	**References**
*SAA3*	26668046–26671801	Chr29: 36.8	12.6	Peak: 24.2	PP	Bagnato et al., *J Dairy Sci, 2008*[33]
*HEATR7B2*	33456348–33528569	Chr20: 37.5	5.6	20.2–43.5 (peak: 31.9)	FP, PP	Arranz et al., *Anim Genet, 1998*[34]
			6.3	37.8–49.7 (peak: 43.8)	PP	Ashwell et al., *J Dairy Sci, 2004*[35]
			0.2	Peak: 37.7	FP, PP	Zhang et al., *Genetics, 1998*[36]
			5.2	15.5–68.0 (peak: 42.7)	PP	Bennewitz et al., *Genetics, 2004*[37]
*TRIB3*	61176217–61187210	Chr13: 75.8	5.2	Peak: 81.0	PP	Bagnato et al., *J Dairy Sci, 2008*[33]
			5.0	Peak: 80.8	FP, PP	Pimentel et al., *Anim Genet, 2011*[38]
*SESN2*	125698159–125714128	Chr2: 116.2	14.6	Peak: 101.6	FP, PP	Pimentel et al., *Anim Genet, 2011*[38]
*HSPD1*	86438978–86449372	Chr2: 81.0	6.5	Peak: 74.5	PP	Bagnato et al., *J Dairy Sci, 2008*[33]
*NR4A1*	27977006–27992869	Chr5: 32.5	12.1	29.4–45.5 (peak: 44.6)	PP	Bennewitz et al., *Genet Sel Evol, 2003*[39]
*SAA1*	26693328–26696498	Chr29: 35.8	11.6	Peak: 24.2	PP	Bagnato et al., *J Dairy Sci, 2008*[33]
*RPL23A*	21526905–21574655	Chr11:27.8	5.8	Peak: 33.6	FP	Ashwell et al., *J Dairy Sci, 1998*[40]
			8.4	Peak: 19.4	PP	Bagnato et al., *J Dairy Sci, 2008*[33]
*VEGFA*	17255946–17269998	Chr23: 25.8	2.5	20.7–41.9 (peak: 28.3)	PP	Viitala et al., *J Dairy Sci, 2003*[41]
			2.5	14.8–42.9 (peak: 28.3)	PP	Elo et al., *Mamm Genome, 1999*[42]
*KRT24*	41557544–41561796	Chr19: 66.1	4.1	Peak: 70.2.	PP	Bagnato et al., *J Dairy Sci, 2008*[33]
			2.2	Peak: 63.9	PP	Mosig et al., *Genetics, 2001*[43]
			4.0	Peak: 62.1	PP	Bennewitz et al., *Genetics, 2004*[37]
			0.8	64.5–69.8 (peak: 66.9)	FP	Bennewitz et al., *Genet Sel Evol, 2003*[39]
*CDKN1A*	10560498–10568780	Chr23: 16.1	12.4	14.8–42.9 (peak: 28.5)	PP	Elo et al., *Mamm Genome, 1999*[42]
*BoLA-DQB*	25375269–25388620	Chr23: 38.2	9.9	14.8–42.9 (peak: 28.3)	PP	Elo et al., *Mamm Genome, 1999*[42]
			9.9	20.7–41.9 (peak: 28.3)	PP	Viitala et al., *J Dairy Sci, 2003*[41]
			4.7	Peak: 42.9	PP	Bagnato et al., *J Dairy Sci, 2008*[33]
*PTHLH*	82246521–82258858	Chr5: 90.7		76.2–103.1	PP	Schrooten et al., *J Dairy Sci, 2004*[44]
			1.1	5.3–104.9 (peak: 89.6)	FP	Bennewitz et al., *Genetics, 2004*[37]
			11.6	34.1–108.3 (peak: 102.3)	PP	Bennewitz et al., *Genetics, 2004*[37]
			5.2	80.1–90.8 (peak: 85.5)	FP	Ashwell et al., *J Dairy Sci, 2004*[35]
*H4*	31665561–31667068	Chr23: 46.8	3.9	Peak: 42.9	PP	Bagnato et al., *J Dairy Sci, 2008*[33]
*DDIT3*	56285007–56307931	Chr5: 61.2	28.4	6.9–102.6 (peak: 89.6)	FP	Bennewitz et al., *Genetics, 2004*[37]
				32.3–69.7	PP	Schrooten et al., *J Dairy Sci, 2004*[44]
*M-SAA3.2*	26755567–26759547	Chr29: 37.8	13.6	Peak: 24.2	PP	Bagnato et al., *J Dairy Sci, 2008*[33]
*HIST1H2AC*	31579046–31589230	Chr23: 46.7	3.8	Peak: 42.9	PP	Bagnato et al., *J Dairy Sci, 2008*[33]

^1^The linkage position was estimated relative to UMD3.1.66 and based on the QTL mapper v.2.019 at http://www.animalgenome.org/cgi-bin/QTLdb/.

PP: protein percentage; FP: fat percentage.

**Table 5:** Detailed information on the nearest and most significant SNPs from previous GWAS to the 31 differentially expressed genes between the mammary gland of two cows with high milk protein and fat percentage and two cows with low protein and fat percentage

**Gene name**	**Gene position (bp)**^**1**^	**Nearest and most significant SNPs of GWAS**			**Traits**	**Raw *p *** **value**	**Reference**
		**Distance**	**Name**	**Position (bp)**^**2**^			
*SAA3*	Chr29: 26668046–26671801	49.5 Kb	ARS-BFGL-NGS-24998	26721324	PP	2.96E-08	Cole et al., *BMC Genomics, 2011*[8]
		4.2 Mb	UA-IFASA-8605	30901735	FP, PP	4.90E-13, 1.50E-16	
*HEATR7B2*	Chr20: 33455667–33528569	23.2 Kb	ARS-BFGL-BAC-2469	33433160	PP	1.21E-08	Jiang et al., *Plos One, 2010*[6]
		2.6 Mb	rs41640170	36097136	PP	1E-6.88	Schopen et al., *J Dairy Sci, 2011*[9]
*TRIB3*	Chr13: 61176217–61187210	2.1 Mb	BFGL-NGS-119420	59101909	FP, PP	1.14E-21, 3.49E-18	Cole et al., *BMC Genomics, 2011*[8]
		9.3 Mb	rs29021058	70524323	PP	0.0018	Kolbehdari et al., *J Anim Breed Genet, 2009*[5]
*SESN2*	Chr2: 125698159–125714128	2.0 Mb	ARS-BFGL-NGS-69013	123717269	FP, PP	1.46E-11, 3.26E-09	Cole et al., *BMC Genomics, 2011*[8]
		2.7 Mb	BTA-31250-no-rs	128437731	FP, PP	3.75E-10, 1.26E-24	
*CHAC1*	Chr10: 36598393–36600764	0.16 Mb	Hapmap53714-rs29017586	36758715	FP, PP	9.84E-07, 6.64E-12	Cole et al., *BMC Genomics, 2011*[8]
*NR4A1*	Chr5: 27977006–27992869	0.99 Mb	ARS-BFGL-NGS-5790	26986116	FP, PP	3.26E-14, 9.43E-28	Cole et al., *BMC Genomics, 2011*[8]
*SAA1*	Chr29: 26693328–26696498	24.8 Kb	ARS-BFGL-NGS-24998	26721324	PP	2.96E-08	Cole et al., *BMC Genomics, 2011*[8]
		4.2 Mb	UA-IFASA-8605	30901735	FP, PP	4.90E-13, 1.50E-16	
*ATF3*	Chr16: 72820025–72832974	1.9 Mb	ARS-BFGL-NGS-70836	74741746	PP	3.58E-07	Cole et al., *BMC Genomics, 2011*[8]
		3.3 Mb	ARS-BFGL-NGS-85980	76091078	FP, PP	6.46E-07, 8.33E-09	
*RPL23A*	Chr11: 21526905–21574655	0.5 Mb	ARS-BFGL-NGS-100459	22128569	PP	1.03E-24	Cole et al., *BMC Genomics, 2011*[8]
		4.0 Mb	BTB-01766447	17484533	FP, PP	4.51E-18, 8.90E-11	
*CDH16*	Chr18: 34733117–34743031	0.75 Mb	ARS-BFGL-NGS-107749	33986486	FP, PP	2.54E-13, 3.55E-18	Cole et al., *BMC Genomics, 2011*[8]
*VEGFA*	Chr23: 17255946–17269998	2.9 Mb	ARS-BFGL-NGS-29490	20166517	FP, PP	4.05E-20, 2.32E-26	Cole et al., *BMC Genomics, 2011*[8]
		1.8 Mb	rs29016156	15422973	PP	0.00099	Kolbehdari et al., *J Anim Breed Genet, 2009*[5]
		0.19 Mb	rs41640789	17061460	FP	0.00012	Kolbehdari et al., *J Anim Breed Genet, 2009*[5]
*BoLA-DQB*	Chr23: 25375269–25388620	4.9 Mb	ARS-BFGL-NGS-72191	30279220	FP, PP	8.46E-17, 1.09E-28	Cole et al., *BMC Genomics, 2011*[8]
*ARID1B*	Chr9: 94882343–95271883	0.37 Mb	ARS-BFGL-NGS-81082	94511747	FP, PP	3.00E-11, 2.37E-08	Cole et al., *BMC Genomics, 2011*[8]
*PTHLH*	Chr5: 82246521–82258858	1.5 Mb	Hapmap51303-BTA-74377	83790390	FP	1.20E-06	Jiang et al., *Plos One, 2010*[6]
		0.63 Mb	ARS-BFGL-NGS-29557	81612132	FP, PP	2.97E-13, 5.97E-25	Cole et al., *BMC Genomics, 2011*[8]
		6.2 Mb	rs41590827	76049666	PP	0.00013	Kolbehdari et al., *J Anim Breed Genet, 2009*[5]
		9.9 Mb	rs41569048	92162494	PP	1E-4.72	Schopen et al., *J Dairy Sci, 2011*[9]
*H4*	Chr23: 31665561–31667068	1.4 Mb	ARS-BFGL-NGS-72191	30279220	FP, PP	8.46E-17, 1.09E-28	Cole et al., *BMC Genomics, 2011*[8]
*FAM71A*	Chr16: 72811910–72814133	2.5 Mb	ARS-BFGL-NGS-85980	76091078	FP, PP	6.46E-07, 8.33E-09	Cole et al., *BMC Genomics, 2011*[8]
*THBS4*	Chr10: 10945424–10999244	0.90 Mb	BTB-00411816	11899342	FP, PP	2.71E-14, 3.45E-25	Cole et al., *BMC Genomics, 2011*[8]
*DDIT3*	Chr5: 56285007–56307931	0.46 Mb	ARS-BFGL-NGS-14781	56766165	FP	5.54E-10	Cole et al., *BMC Genomics, 2011*[8]
*M-SAA3.2*	Chr29: 26755567–26759547	34.2 Kb	ARS-BFGL-NGS-24998	26721324	PP	2.96E-08	Cole et al., *BMC Genomics, 2011*[8]
		4.1 Mb	UA-IFASA-8605	30901735	FP, PP	7.90E-13, 1.50E-16	
*HIST1H2AC*	Chr23: 31579046–31589230	0.95 Mb	BTA-68781-no-rs	30711619	FP, PP	1.17E-17, 6.08E-27	Cole et al., *BMC Genomics, 2011*[8]
*P4HA2*	Chr7: 23480314–23513315	1.1 Mb	Hapmap49309-BTA-78604	24655689	PP	4.34E-14	Cole et al., *BMC Genomics, 2011*[8]

^1,2^Means the position on the bovine genome sequence of UMD3.1.66.

PP: protein percentage; FP: fat percentage.

Among the 31 differentially expressed genes, 17 were found to be located within QTL regions that were confirmed to have large genetic effects on milk PP and FP, including *SAA3* (12.6 cM), *HEATR7B2* (0.2 ~ 6.3 cM), *TRIB3* (5.0 ~ 5.2 cM), *SESN2* (14.6 cM), *HSPD1* (6.5 cM), *NR4A1* (12.1 cM), *SAA1* (11.6 cM), *RPL23A* (5.8 ~ 8.4 cM), *VEGFA* (2.5 cM), *KRT24* (0.8 ~ 4.1 cM), *CDKN1A* (12.4 cM), *BoLA-DQB* (4.7 ~ 9.9 cM), *PTHLH* (1.1 ~ 11.6 cM), *H4* (3.9 cM), *DDIT3* (28.4 cM), *M*-*SAA3.2* (13.6 cM), and *HIST1H2AC* (3.8 cM).

On the other hand, 21 genes were found to be within 23.2 Kb to 9.9 Mb of one or multiple significant SNPs for milk PP and FP detected in previous GWASs in dairy cattle (Table 5).

Combining the QTL and GWAS data, 14 genes were near to both the peak locations of the reported QTLs and significant SNPs for PP and FP, including *SAA3*, *HEATR7B2*, *TRIB3*, *SESN2*, *NR4A1*, *SAA1*, *RPL23A*, *VEGFA*, *BoLA-DQB*, *PTHLH*, *H4*, *DDIT3, M*-*SAA3.2*, and *HIST1H2AC*.

## Discussion

In this study, we investigated the whole genome transcriptome profile of the bovine mammary epithelium in Holstein cows using RNA sequencing (RNA-seq), with the aim of identifying candidate genes for milk composition traits at a finer resolution. RNA-seq has many advantages over traditional cDNA microarray technologies: it is free from probe design issues or bias from hybridization issues [14] and easily detects low-abundance genes [15]. Marioni et al. reported that the Pearson correlation between RNA-Seq and qRT-PCR could reach 0.929, suggesting that the RNA-Seq technique is accurate and reproducible [15]. RNA-seq is the most powerful tool available for the deep research of the complexity of the transcriptome.

In our analysis, to minimize false-positive errors and ensure substantial detection power and accuracy, several strategies were applied to detect the differentially expressed genes between milking Holstein cows with high PP and FP and cows with low PP and FP, by controlling the critical influencing factors. We sequenced the transcripts deeply (5–10G of data per transcriptome), and performed the analysis using two commonly used package: DESeq and Cuffdiff. Only those differentially expressed genes ranked in the top half of the expressed genes were considered. By contrast, the differentially expressed genes expressed in the bottom half level were rooted out, as suggested by Rapaport et al. [30]. Of note, in our study design, only two biological replicates were used for each condition, because of the limited budget and sample availability. Rapaport et al. (2013) [30] investigated the impact of different sequencing depths and varying number of replicates on the identification of differentially expressed genes. They demonstrated that with most methods, over 90% of differently expressed genes at the top expression levels could be detected with using two replicates and 5% of the reads [30]. However, more biological replications are still preferred and recommended to ensure broader application [30]. The more replicates are taken, the more the detection power is improved.

We identified 31 significantly differentially expressed genes whose mRNA levels changed between the Holstein cows with extremely high and low PP and FP, respectively (*p* < 0.05, FDR *q* < 0.05). Among them, 14 genes were not only located within the known QTL regions for PP and FP, but were also close to significant SNPs for these traits detected by one or more independent previous GWASs [5,6,8,9], such as *SAA3*, *HEATR7B2*, *TRIB3*, *SESN2*, *NR4A1*, *SAA1*, *RPL23A*, *VEGFA*, *BoLA-DQB*, *PTHLH*, *H4*, *DDIT3, M*-*SAA3.2*, and *HIST1H2AC*.

Tribbles homolog 3 (*TRIB3*), a pseudokinase, plays a critical role in the regulation of several signaling pathways involved in cell survival and/or cell stress, and inhibits phosphorylation of AKT/protein kinase B (AKT), which is an important enzyme in the signal transduction of growth factor receptors [45,46]. It is thought that TRIB3 can stimulate lipolysis by promoting ubiquitination and degradation of acetyl-coenzyme A carboxylase (ACC), which is a key regulatory enzyme in the fatty acid synthesis pathway in adipose tissue, through an association with the E3 ubiquitin ligase constitutive photomorphogenic protein 1 (COP1), an adaptor protein [47]. Hence, TRIB3 was suggested to negatively regulate fat synthesis. This evidence is consistent with the expression profile of *TRIB3* gene in this study. As shown in Table 1, the mRNA level of *TRIB3* in the cows with a low FP is higher than that in cows with a higher FP (*p* < 0.01, *q* < 0.01). Our IPA pathway analysis also showed that *TRIB3* gene was involved in lipid metabolism. More importantly, *TRIB3* was near to the peak location of two reported QTLs for milk PP and FP and two significant SNPs for PP and FP [5,8]. Taken together, the analyses indicate that *TRIB3* is one of the most promising candidate genes for milk FP in dairy cattle.

Vascular endothelial growth factor A (VEGFA) is a heparin-binding growth factor specific for vascular endothelial cells, with a potent angiogenic capacity, both in physiological and pathological conditions [48]. VEGF has an essential regulatory role in the secretory and immunological functions of the mammary gland, such as angiogenesis, vascular permeability, and migration of mononuclear leukocytes [48]. Alkafafy (2005) found that VEGF-immunoreactive mast cells release a variety of angiogenic factors in the bovine mammary gland epididymal interstitium [49]. TR3/NR4A1, also a differentially expressed gene in this study, is the transcription factor that regulates VEGFA-mediated angiogenesis [50]. Integrated analysis revealed that *VEGFA* was near to the peak positions of two QTLs for PP, and very close to significant SNPs for PP and FP detected in two previous GWAS reports [5,8], one of which was ranked in the top 100 most significant SNPs by Cole et al. [8]. Thus, *VGEFA* may be a promising candidate gene for milk PP and FP.

Parathyroid hormone-like hormone (PTHLH), also known as parathyroid hormone-related protein (PTHrP), was first identified as a parathyroid hormone (PTH)-like factor responsible for humoral hypercalcemia in malignancies [51]. Similar evidence indicated that bovine mammary *PTHLH* is closely related to the Ca concentration in milk [52]. *PTHLH* is expressed in the mammary gland and appears to be critical for the morphogenesis and angiogenesis of this structure, and its production in the mammary gland is generally attributed to epithelial cells [53]. Reduction of *PTHLH*’s expression to very low levels prevented mammary gland development, and genetic disruption of this gene results in a failure of mammary development in mice and humans [54]. Similar to *TRIB3* and *VEGFA* genes, *PTHLH* is not only close to the PP and FP of QTLs but also is near to significant SNPs that were detected by four independent GWASs [5,6,8,9]. Therefore, *PTHLH* also represents a candidate gene for milk PP and FP. A previous study showed a similar result: *PTHLH* was differentially expressed between before parturition and in early lactation in Holstein dairy cows [22].

*SAA* is a multigene family comprising four genes (*SAA1–4*) that may play a direct physiological role in local and systemic inflammation, and free fatty acid production and cholesterol metabolism. SAA is an apolipoprotein and a component of high-density lipoprotein (HDL) particles [55]. Serum amyloid A3 (SAA3) participates in lipid metabolism, cholesterol transport, and apoptosis of mammary epithelial cells [56-58]. Several previous studies showed that the mRNA level of *SAA3* changed across the different periods of mammary gland development and lactation in cows, horses, sheep, and goats [59,60]. Bovine *M-SAA3* was differentially expressed by the mammary epithelium in response to PRL, which regulates a wide spectrum of physiological processes, such as mammary gland development, lactation, and immune function; thus, *M-SAA3* has an important tissue-specific function during lactation and mammary infection [61]. Furthermore, *SAA1*, *SAA3*, and *M-SAA3* are located within the QTL region for PP and near to a significant SNP for PP and FP (Tables 4 and 5). *SAA1* was also shown to be involved in lipid metabolism by IPA pathway analysis.

There are only a few reports regarding *ribosomal protein L23A* (*RPL23A*), *histone H4* (*H4*), and *histone cluster 1, H2ac* (*HIST1H2AC*). RPL23A plays a very important role in translation and participates in extra-ribosomal functions, such as apoptosis, cell division, and differentiation [62-64]. Histone H4 is one of four core histones (H2A, H2B, H3, and H4), which are basic nuclear proteins and are responsible for the nucleosome structure of the chromosomal fiber in eukaryotes. *HIST1H2AC* is located in the large histone gene cluster on BTA23. Although the two genes were close to a QTL for milk PP and near to the most significant SNPs for milk PP and FP [8], to date, little is known as to whether the two genes are associated with quantitative traits such as milk production traits. Histone acetylation is an important epigenetic modification. Modifications of lysine residues of core histones have the greatest potential for unfolding chromatin to recruit different transcriptional factors, which is almost invariably associated with activation of gene transcription [65]. Our data implied that epigenetic regulation might be involved in milk production and mammary gland development through histone modifications.

In addition, integrated analysis of differential expression patterns, QTL and GWAS evidence, and biological functions revealed that five other genes, namely *SESN2, NR4A1, DDIT3, HEATR7B2*, and *BoLA-DQB*, were also associated with milk composition traits to some extent. These five genes are located near the peak positions of known QTLs and several significant SNPs for milk PP and FP (Tables 4 and 5). Sestrin 2 (SESN2) is involved in DNA damage and genetic instability [66]. Deficiency of *SESN2* in some cell lines resulted in growth inhibition instead of growth stimulation [67]. NR4A1 belongs to the NR4A family of nuclear receptors, and is considered a mediator/regulator of cell survival and apoptosis in breast cancer cells; it is up-regulated in primary breast cancer compared with normal tissue [68]. NR4A receptors act as transcription factors, altering the expression of downstream genes, e.g. *VEGF*, in angiogenesis, proliferation, DNA repair, metabolism, and cell migration [69]. DNA-damage-inducible transcript 3 protein (DDIT3) is a member of the leucine zipper transcription factor family, which is implicated in adipocyte differentiation [70]. ATF3 belongs to the mammalian activation transcription factor/cAMP responsive element-binding (CREB) protein family of transcription factors. It is generally viewed as a transcriptional repressor and represses the transcription of *DDIT3*[71,72]. GO and IPA analysis indicated that both *DDIT3* and *ATF3* are involved in accumulation of glycoproteins, apoptosis, and regeneration of epithelial cells. There is no information available for *HEATR7B2* (*maestro heat-like repeat family member 2B*). BoLA-DQB is one of the major histocompatibility complex (MHC) class II molecules that play a central role in the regulation of the immune response. Generally, it is thought that *MHC* genes affect production traits indirectly by increasing the overall disease resistance of the individual. Bionaz et al. also found that the expression of *MHC class I* molecules decreased from the beginning of lactation to 120 days compared with 230 days in cows [23]. The mammary gland is actively involved in the immune system and is an evolutionary product of the innate immune system. The MHC pathway is a vesicle-dependent process, which uses ER-Golgi networks, as do milk components; thus, the MHC can be considered a competitor of the vesicle-transport system, which may explain its deceased activity implied herein [23].

Previous studies on bovine milk and mammary transcriptomes over various lactation periods revealed several metabolic pathways related to bovine mammary and lactation, such as energy synthesis, lipid metabolism, protein synthesis, cell cycle/death, angiogenesis, immune function, and epigenetic regulation [18,20-23]. Our results were consistent with these reports (Tables 1, 2 and 3).

The interpretation of the findings from the present study still has limitations. In cow’s milk, there are two major protein groups: caseins (αS1-casein, αS2-casein, β-casein, and κ-casein) and whey proteins (α -lactalbumin and lactoglobulin). However, our study did not show significant changes in milk protein mRNA expression. Similar results were observed by previous investigations, which showed that none of the milk protein genes (caseins, α-lactalbumin, and β-lactoglobulin) showed significant changes in expression from late pregnancy to early lactation [22], and in the lactating mammary gland compared with the non-lactating mammary gland [73] in Holstein dairy cows. Such a phenomenon may be explained if the expression of milk protein genes reaches a plateau in late lactation [22]. *DGAT1* and *Jak-STAT* genes, with large effect on milk traits, were not differentially expressed between the high and low groups in this study. Such a result is similar to those reported by Finucane et al. [22] and Buinaz et al. [23]. In addition, our RNA-seq data indicated that 28 of the 31 differentially expressed genes were downregulated in the cows with higher PP and FP compared with the low group. Although it is reasonable to expect that lactation requires increased expression or turning-on of many genes, the same result was observed in a previous bovine mammary transcriptome analysis, which found that more than twice as many genes were downregulated than upregulated in early lactation compared with non-lactation (before parturition) in Holstein dairy cows [22].

Currently, genomic selection is the main application used in animal breeding in dairy cattle, in which the Illumina 50 K chip and GeneSeek 90 K chip are commonly used. Most of the SNP markers in these two types of chips were collected from the current SNP database and are almost evenly distributed across the whole genome. Thus, certain major genes with large genetic effects on one or milk milk traits could be put into the SNP chip instead of used in marker/gene-assisted selection to increase selection efficiency in some specific dairy cattle populations.

## Conclusions

The present study provided a global view of the complexity of the bovine mammary epithelium cell transcriptome, and revealed 31 differentially expressed genes between Holstein cows with extremely high and low milk PP and FP. Integrated analysis of differential gene expression, QTL and GWAS data, and biological functions suggested that seven genes, including *TRIB3*, *SAA* (*SAA1*, *SAA3*, and *M-SAA3.2*), *VEGFA, PTHLH*, and *RPL23A*, represent the most promising candidates affecting milk PP and FP of dairy cattle.

## Abbreviations

QTL: Quantitative trait locus; LA: Linkage analysis; LD: Linkage disequilibrium; GWAS: Genome-wide association study; DGAT1: AcylCoA: diacylglycerol acyltransferase; GHR: Growth hormone receptor; NGS: Next generation sequencing; RNA-seq: RNA-sequencing; MY: Milk yield; FY: Fat yield; PY: Protein yield; FP: Fat percentage; PP: Protein percentage; DHI: Dairy herd improvement; DAC: Dairy association of China; RIN: RNA integrity number; FRKM: Fragments per kilobase of transcript per million mapped fragments; GO: Gene ontology; IPA: Ingenuity pathways analysis; qRT-RCP: Real-time quantitative reverse-transcription polymerase chain reaction; TRIB3: Tribbles homolog 3; PTHLH: Parathyroid hormone-like hormone; ARID1B: AT rich interactive domain 1B (SWI1-like); ZC3H14: Zinc finger CCCH-type containing 14; H4: Histone H4; DDIT3: DNA-damage-inducible transcript 3; CHAC1: Cation transport regulator homolog 1; SAA3: Serum amyloid A3; ATF3: Activating transcription factor 3; HEATR7B2: HEAT repeat family member 7B2; SESN2: Sestrin 2; NR4A1: Nuclear receptor subfamily 4, group A, member 1; SAA1: Serum amyloid A1; RPL23A: Ribosomal protein L23a; VEGFA: Vascular endothelial growth factor A; THBS4: Thrombospondin 4; CSN3: K-casein; CSN2: β-casein; LALBA: a-lactalbumin; MBLG: β-lactoglobulin; STAT5A: Signal transducer and activator of transcription5A; STAT5B: Signal transducer and activator of transcription5B; SCD: Stearoyl-coenzyme A desaturase; KRT24: Keratin 24; EIF4G3: Eukaryotic translation initiation factor 4 gamma, 3; CDKN1A: Cyclin-dependent kinase inhibitor 1A; GAD2: Glutamate decarboxylase 2; FDR: False discovery rate; ACC: Acetyl–coenzyme A carboxylase; COP1: Constitutive photomorphogenic protein 1; PTHrP: Parathyroid hormone-related protein; H: Histone; CREB: cAMP responsive element-binding; BTA: Bos Taurus autosome; SNP: Single nucleotide polymorphism.

## Competing interests

The authors declare that they have no competing interests.

## Authors’ contributions

XC performed the RNA-related experiments, data analysis, and prepared the manuscript. YH contributed to the bioinformatics analysis of the sequencing data. SY and YX collected the samples and performed the RNA experiments. SZ, QZ, and YZ participated in the experimental design, result interpretation, and sample collection. XL participated in the data analysis. GEL performed the gene function and pathway analysis. DS conceived and designed the experiments, and prepared the manuscript. All authors read and approved the final manuscript.

## Supplementary Material

Additional file 1: Figure S1 Genotypes of DGAT1 p.Lys232Ala mutation for four cows detected by PCR product sequencing. Arrowhead indicates the two nucleotides of DGAT1 p.Lys232Ala mutation. Click here for file

Additional file 2: Table S1 The basic statistics for RNA-seq reads generated from mammary glands of two cows with high milk protein and fat percentage and two cows with low protein and fat percentage, and the subsequent alignment information with Tophat^1^. Click here for file

Additional file 3: Table S2 PCR primers for qRT-PCR validation of 11 differentially expressed genes between the mammary gland of two cows with high milk protein and fat percentage and two cows with low protein and fat percentage. Click here for file

Additional file 4: Figure S2 Correlation between biological replicates within two cows with high milk PP and FP and two cows with low PP and FP. The x- and y-axis correspond to the FPKM value of each sample. High and low in the x- and y-axis mean two cows with high milk PP and FP and two cows with low PP and FP, respectively. The correlation coefficient (R^2^) between two individuals within each group was calculated based on FPKM value of each individual. R^2^ was used to evaluate the read similarity and reliability of biological replicates within group. Click here for file

Additional file 5: Figure S3 Comparison of the expression ratios of 11 randomly chosen differentially expressed genes between the mammary glands of two cows with high milk PP and FP and two cows with low PP and FP using RNA-seq and qRT-PCR. A. Black and gray columns represent the relative mRNA expression levels by qRT-PCR normalized by *GAPDH* and *ACTB*, respectively; white columns show the *log*_*10*_ (fragments per kilobase of transcript per million fragments mapped; FPKM) value obtained by RNA-seq. High and low in the x-axis mean cows with high and low PP and FP, respectively. B-1. The *x*- and *y*-axis show the log2 (ratio of mRNA levels, low/high) measured by qRT-PCR (normalized by *GAPDH*) and RNA-seq, respectively. B-2. The *x*- and *y-*axis shows the log2 (ratio of mRNA levels, low/high) measured by qRT-PCR (normalized by *ACTB*) and RNA-seq, respectively. Click here for file
