# Supplementary material for: Transcriptional profiling of mammary gland in Holstein cows with extremely different milk protein and fat percentage using RNA sequencing
Source: BMC Genomics. 2014 Mar 24;15:226. doi: 10.1186/1471-2164-15-226 (PMC3998192; doi:10.1186/1471-2164-15-226)
Supplement: Additional file 1: Figure S1 — Genotypes of DGAT1 p.Lys232Ala mutation for four cows detected by PCR product sequencing. Arrowhead indicates the two nucleotides of DGAT1 p.Lys232Ala mutation. [file 1471-2164-15-226-S1.doc]

**A：cow1 (KA genotype)**


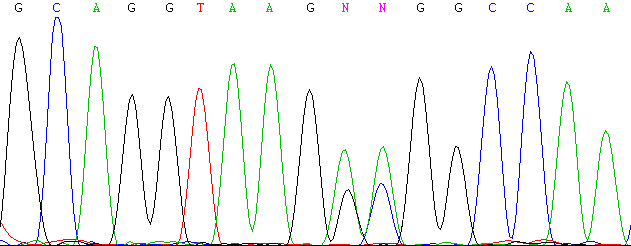


**B：cow2 (KA genotype)**


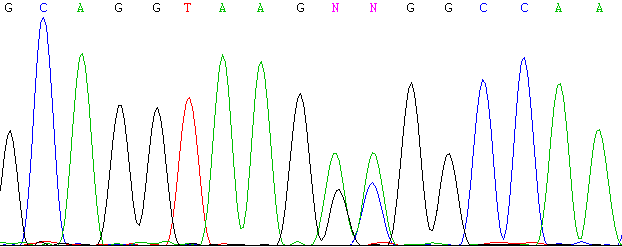


**C：cow3 (KA genotype)**


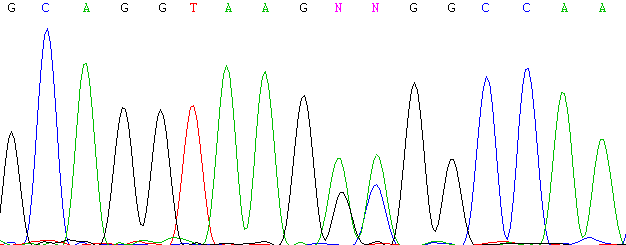


**D：cow4 (AA genotype)**


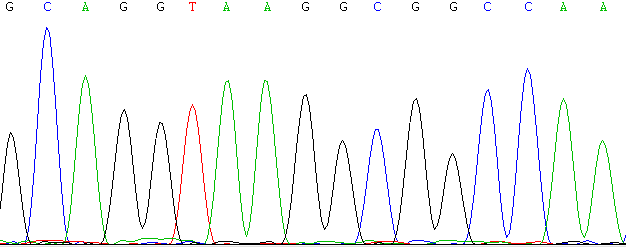


**Additional file 1: Figure S1. Genotypes of DGAT1 p.Lys232Ala mutation for four cows detected by PCR product sequencing.**

Arrowhead indicates the two nucleotides of DGAT1 p.Lys232Ala mutation.
