# Supplementary material for: Transcriptional profiling of mammary gland in Holstein cows with extremely different milk protein and fat percentage using RNA sequencing
Source: BMC Genomics. 2014 Mar 24;15:226. doi: 10.1186/1471-2164-15-226 (PMC3998192; doi:10.1186/1471-2164-15-226)
Supplement: Additional file 2: Table S1 — The basic statistics for RNA-seq reads generated from mammary glands of two cows with high milk protein and fat percentage and two cows with low protein and fat percentage, and the subsequent alignment information with Tophat1. [file 1471-2164-15-226-S2.doc]

**Additional file 2: Table S1. The basic statistics for RNA-seq reads generated from mammary glands of two cows with high milk protein and fat percentage and two cows with low protein and fat percentage, and the subsequent alignment **information with Tophat^1^.****

| Mapping summary | cow1  (high1) | cow2  (high2) | cow3  (low1) | cow4  (low2) |
| --- | --- | --- | --- | --- |
| Total reads (raw reads) | 53,294,906 | 73,924,198 | 57,911,722 | 83,019,642 |
| Clean reads | 53,246,940 | 73,865,058 | 57,865,392 | 82,944,924 |
| Statistic for clean reads: Q20 | 97.84% | 97.86% | 97.86% | 97.91% |
| Statistic for clean reads: Q30 | 94.38% | 94.45% | 94.39% | 94.51% |
| Statistic for clean reads: GC content | 47.08% | 46.40% | 46.49% | 46.44% |
| Statistic for clean reads: Seq-Dupl-level | 81.58% | 86.73% | 86.79% | 83.69% |
| Statistic for clean reads: Error rate | 0.025% | 0.025% | 0.025% | 0.025% |
| Total mapped reads | 49,619,209 | 68,027,129 | 53,501,482 | 76,963,998 |
| Unique mapped reads | 48,967,376 | 66,997,952 | 52,113,820 | 75,572,578 |
| Multiple mapped reads | 651,833 | 1,029,177 | 1,387,662 | 1,391,420 |
| Junction mapped reads | 25,836,411 | 38,556,766 | 29,584,413 | 40,364,410 |
| Mapping rate | 93.10% | 92.02% | 92.38% | 92.71% |

^1^Bovine genome UMD3.1.66 was used for the alignment.

Q20: **the proportion of bases with a** phred base quality score greater than 20; i.e., the proportion of read bases whose error rate is less than 1%

Q30: **the proportion of bases with a** phred base quality score greater than 30; i.e., the proportion of read bases whose error rate is less than 0.1%.

Seq-Dupl-level: **sequence duplication level.**
