# Supplementary material for: Transcriptional profiling of mammary gland in Holstein cows with extremely different milk protein and fat percentage using RNA sequencing
Source: BMC Genomics. 2014 Mar 24;15:226. doi: 10.1186/1471-2164-15-226 (PMC3998192; doi:10.1186/1471-2164-15-226)
Supplement: Additional file 3: Table S2 — PCR primers for qRT-PCR validation of 11 differentially expressed genes between the mammary gland of two cows with high milk protein and fat percentage and two cows with low protein and fat percentage. [file 1471-2164-15-226-S3.doc]

**Additional file 3: Table S2. PCR primers for qRT-PCR validation of 11 differentially expressed genes between the mammary gland of two cows with high milk protein and fat percentage and two cows with low protein and fat percentage.**

| Gene name | Fold change  by RNA-seq | Amplicon content | Forward primer sequence | Reverse primer Sequence | Amplicon (bp) | Tm (°C) |
| --- | --- | --- | --- | --- | --- | --- |
| *SAA3* | −2.09 | exon4 | CCAACCACTTCAGACCTGCT | GCTGCCTTCTGAGGACAGAG | 131 | 59 |
| *TRIB3* | −2.64 | exon4 | ATCCACTCGTGCTGATACCC | GGGCTCTAGGGTCCTGAATC | 157 | 59 |
| *SESN2* | −3.01 | exon9 | CCAGCAGGTCTGAAGTGGTT | aatgacctgcccaagatcac | 164 | 59 |
| *CHAC1* | −2.86 | exon3 | AGATCATGAGGGCTGCACTT | AATGCCTTGAGTGGTTGGTC | 165 | 59 |
| *SAA1* | −5.84 | exon3, exon4 | AGCCAACTACAAGGATGCAGA | CCAGCAGGTCTGAAGTGGTT | 246 | 59 |
| *ATF3* | −2.70 | exon4 | TACATCCGAAACCCTGAAGC | TCTGGAGAATCCAAGGATGG | 214 | 59 |
| *ARID1B* | −0.70 | exon11, exon12 | CCGACTCTATGTCTGCGTCA | TTGCACTCAAAGGCAAACAG | 162 | 59 |
| *PTHLH* | −0.76 | exon3 | CACGGAGGCATTGAATTTTT | TAGAGCAATGGGGGAGACAG | 149 | 59 |
| *ZC3H14* | −0.65 | exon8, exon9, exon10 | CACTCACATGAGCCGAAGAA | CACTCACATGAGCCGAAGAA | 187 | 59 |
| *H4* | −2.17 | exon1 | ACCGCTCTAGTGGGAAGGAT | CCTGGAAAATTCGAAGGACA | 163 | 59 |
| *DDIT3* | −1.70 | exon2, exon3 | TGGAAGCCTGGTATGAGGAC | GGGAGGTGTGTGTGACCTCT | 177 | 59 |
| *GAPDH* |  | exon2, exon3, exon4 | AGATGGTGAAGGTCGGAGTG | CGTTCTCTGCCTTGACTGTG | 189 | 59 |
| *ACTB* |  | exon4, exon5 | ctcttccagccttccttcct | gggcagtgatctctttctgc | 138 | 59 |
