# Supplementary material for: Transcriptional profiling of mammary gland in Holstein cows with extremely different milk protein and fat percentage using RNA sequencing
Source: BMC Genomics. 2014 Mar 24;15:226. doi: 10.1186/1471-2164-15-226 (PMC3998192; doi:10.1186/1471-2164-15-226)
Supplement: Additional file 4: Figure S2 — Correlation between biological replicates within two cows with high milk PP and FP and two cows with low PP and FP. The x- and y-axis correspond to the FPKM value of each sample. High and low in the x- and y-axis mean two cows with high milk PP and FP and two cows with low PP and FP, respectively. The correlation coefficient (R2) between two individuals within each group was calculated based on FPKM value of each individual. R2 was used to evaluate the read similarity and reliability of biological replicates within group. [file 1471-2164-15-226-S4.doc]

**
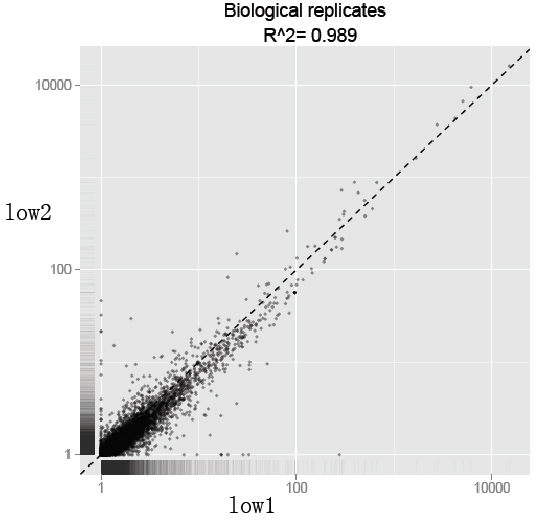

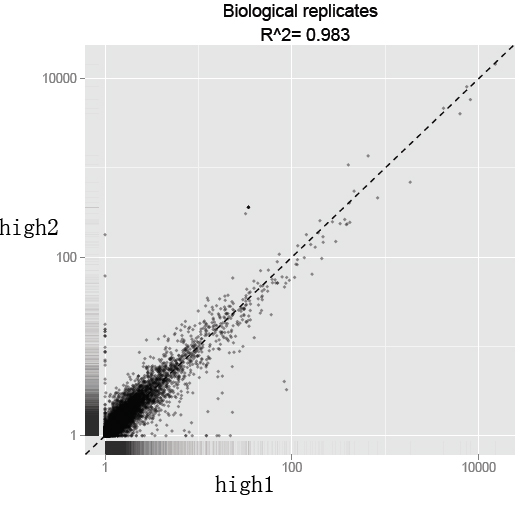
**

**Additional file 4: Figure S2. Correlation between biological replicates within two cows with high milk PP and FP and two cows with low PP and FP.**

The x- and y-axis correspond to the FPKM value of each sample. High and low in the x- and y-axis mean two cows with high milk PP and FP and two cows with low PP and FP, respectively. The correlation coefficient (R^2^) between two individuals within each group was calculated based on FPKM value of each individual. R^2^ was used to evaluate the read similarity and reliability of biological replicates within group.
