# Supplementary material for: Transcriptional profiling of mammary gland in Holstein cows with extremely different milk protein and fat percentage using RNA sequencing
Source: BMC Genomics. 2014 Mar 24;15:226. doi: 10.1186/1471-2164-15-226 (PMC3998192; doi:10.1186/1471-2164-15-226)
Supplement: Additional file 5: Figure S3 — Comparison of the expression ratios of 11 randomly chosen differentially expressed genes between the mammary glands of two cows with high milk PP and FP and two cows with low PP and FP using RNA-seq and qRT-PCR. A. Black and gray columns represent the relative mRNA expression levels by qRT-PCR normalized by GAPDH and ACTB, respectively; white columns show the log10 (fragments per kilobase of transcript per million fragments mapped; FPKM) value obtained by RNA-seq. High and low in the x-axis mean cows with high and low PP and FP, respectively. B-1. The x- and y-axis show the log2 (ratio of mRNA levels, low/high) measured by qRT-PCR (normalized by GAPDH) and RNA-seq, respectively. B-2. The x- and y-axis shows the log2 (ratio of mRNA levels, low/high) measured by qRT-PCR (normalized by ACTB) and RNA-seq, respectively. [file 1471-2164-15-226-S5.doc]

**A.**


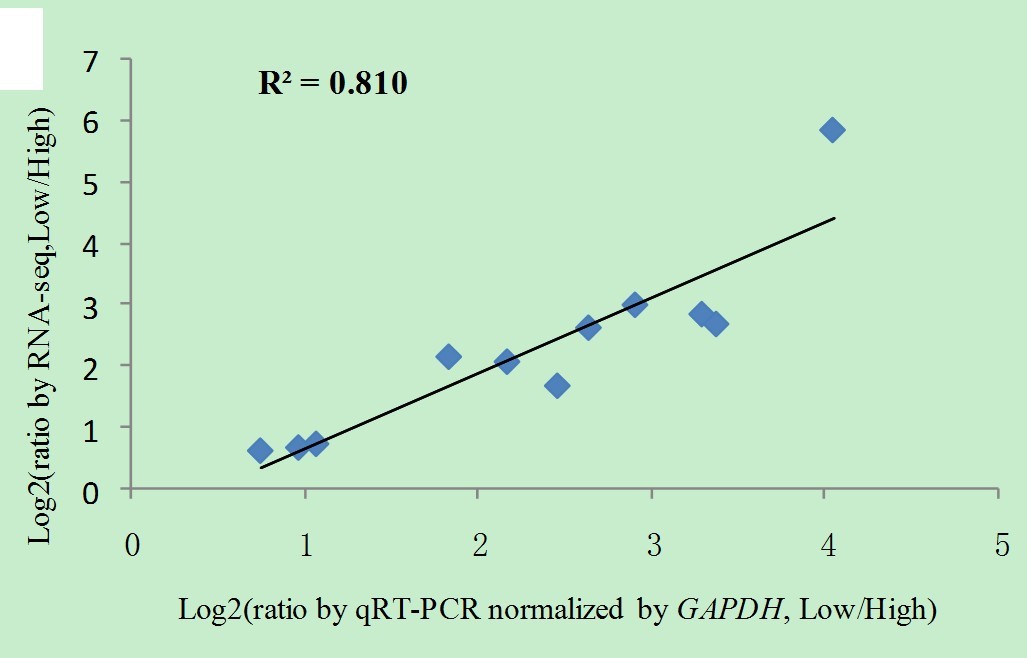


**B-1.**


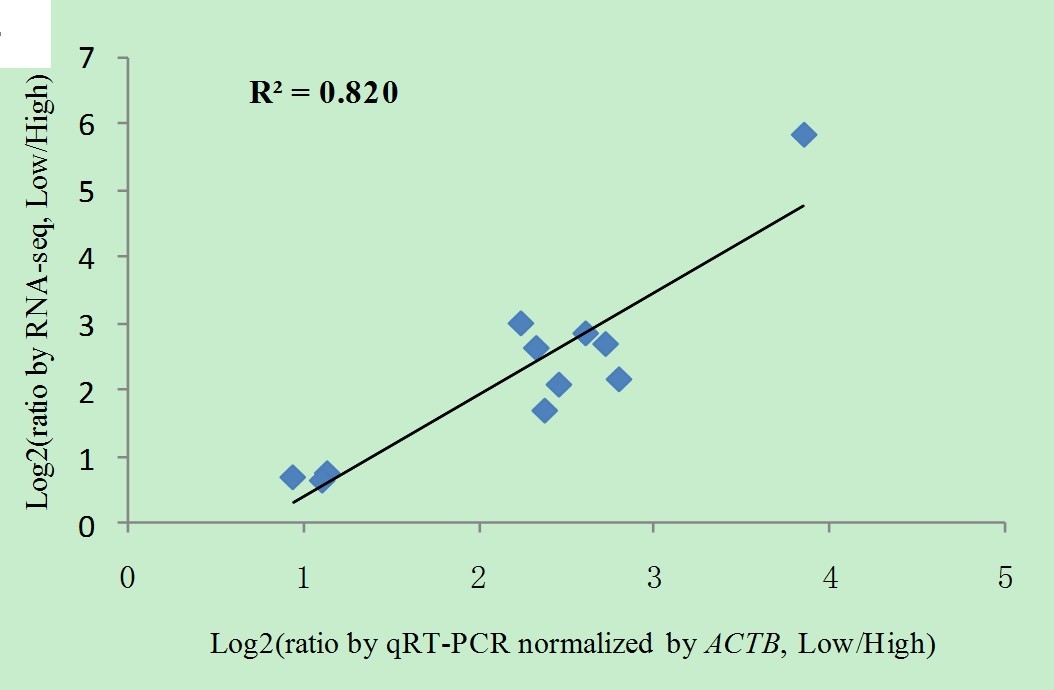


**B-2.**

**Additional file 5: Figure S3. Comparison of the expression ratios of 11 randomly chosen differentially expressed genes between the mammary glands of two cows with high milk PP and FP and two cows with low PP and FP using RNA-seq and qRT-PCR.**

**A.** Black and gray columns represent the relative mRNA expression levels by qRT-PCR normalized by *GAPDH* and *ACTB*, respectively; white columns show the *log_10_* (fragments per kilobase of transcript per million fragments mapped; FPKM) value obtained by RNA-seq. High and low in the x-axis mean cows with high and low PP and FP, respectively.

**B-1**. The *x*- and *y*-axis show the log2 (ratio of mRNA levels, low/high) measured by qRT-PCR (normalized by *GAPDH*) and RNA-seq, respectively.

**B-2.** The *x*- and *y-*axis shows the log2 (ratio of mRNA levels, low/high) measured by qRT-PCR (normalized by *ACTB*) and RNA-seq, respectively.
